# National Health Insurance Notes and Comments

**Published:** 1922-11

**Authors:** 


### NATIONAL HEALTH INSURANCE NOTES AND COMMENTS.

**The Scope of Medical Service for the Insured.**—The following resolution, proposed to be moved by the City of Worcester Insurance Committee at the Annual Conference of the National Association of Insurance Committees, has attracted a good deal of publicity :—

That in the opinion of this meeting the total funds now contributed by insured persons, their employers and the State, should be regarded as sufficient to provide for the inclusion in " Medical Benefit " of all kinds of medical and surgical treatment (comprising major operations, specialist and consultant services, hospital facilities, laboratory diagnosis, &c., &c.) ; the cost of any service other than that usually rendered by a practitioner of ordinary professional competence and skill to be a first charge on the Practitioners' Fund ; and that the Medical Benefit Regulations and any schemes made thereunder should be amended accordingly.

In his address to the Association, which has been fully reported in the " National Insurance Gazette " for October 14, 1922, Mr. Brock, the Assistant Secretary to the Ministry of Health responsible for the arrangements for Medical Benefit, stated that the cost of such an extension of medical services for the insured was extraordinarily difficult to estimate ; when the question was examined in consultation with many leaders of the medical profession in 1919 and 1920 a most conservative estimate placed the probable cost at one-third of the amount then being spent on medical benefit. This, he thought, was an underestimate. The Worcester resolution surely stands, however, condemned in any case, as implying an altogether wrong method of dealing with the subject. Who can say that existing funds are adequate for a service costing $x$ million pounds ? Is it to be supposed that the doctors engaged in insurance practice will consent to take what is left after $x$ million pounds have been taken out of the funds available for medical benefit ? It is probable that questions of this sort would have been answered if the resolution had been proceeded with ; it was, in fact, withdrawn.

**Laboratory Services.**—A more practical proposal in the present condition of finances was that embodied in the resolution drafted by the Executive Committee of the National Association of Insurance Committees :—

That the resources of the medical and allied sciences, including radiology, bacteriology and biochemistry should be available to insurance practitioners in the diagnosis of the diseases and the treatment of insured persons, and that such services should form part of medical benefit.

If the estimate of the West Riding Insurance Committee, put forward a month or two ago, that a complete laboratory service would cost little more than 1d. per insured person per annum is a reliable guide, there is certainly here a proposal of immediate practical interest. The above resolution was moved by Sir Wm. Glyn-Jones, the president-elect of the Association, who said that if medical treatment was to be fully effective the panel practitioners should have the whole resources of laboratory service in order that treatment should be accurately diagnosed. Millions of insured persons were receiving better treatment than they had in pre-Insurance Act days. But still the treatment fell far below what they had a right to expect. When Government representatives talked about the cost of providing aids to a proper medical service, he could not help thinking that the Governments of Europe and America a few years ago paid very large sums to the scientists and research chemists for finding out something that would kill. His proposal was for saving life, and the money which had been spent in providing material to kill would have maintained the service he had proposed for the next 100 years.

The motion was carried.

**Investigation of Excessive Prescribing.**—In a paper read to the National Association of Clerks to Insurance Committees at their Annual Conference held at Southend-on-Sea on October 7, Mr. Lee Gordon, the Clerk to the Coventry Insurance Committee, put forward for discussion some proposals for a change of machinery in connection with the investigation of the cost of doctors' prescribing for his insured patients. He stated that very strong grounds existed for doubting whether more than half the Panel Committees conduct the duties laid upon them under Article 34 of the Medical Benefit Regulations in such a manner as to result in the effective determination of the question whether a practitioner was prescribing extravagantly, or in the penalisation of such a doctor; while, he said, it was undoubtedly a fact that in many cases doctors who could not possibly be excused of unnecessarily costly prescribing had been deterred from ordering what they would honestly consider the best thing for their patients, for fear of bringing their prescribing under the notice of their Panel Committee. (If such doctors are so deterred by the fact that prescribing in excess of a certain cost is automatically brought under review, we fail to find anything in the proposals made by Mr. Lee Gordon, which would alter this).

Mr. Gordon appears to be perturbed by the announcement which has been made that the Regional Medical Staff of the Ministry are to be associated with the Insurance Committees and Panel Committees in the investigation of excessive prescribing. He infers either that this medical staff will do the work in default of the Panel Committee, or that the Minister contemplates introducing new methods of scrutiny by his own Department with some nominal co-operation with the Panel Committee and association with the Insurance Committee, and he sees in the proposal the possibility of a further undermining of the Insurance Committees' administrative status. It therefore, he says, behoves Insurance Committees to be up and doing, especially as it is thought that Panel Committees have been discouraged by a decision of the Ministry in allowing the appeal of a practitioner against the surcharge of an Insurance Committee in a case which had obviously involved a good deal of time in its preparation.

In these circumstances, his proposal is that, as the Insurance Committee has standing facilities and staff trained in administrative work, this could be put at the disposal of a sub-committee of the Insurance Committee, especially constituted to investigate all questions of prescribing, to consist of two members who should be practitioners appointed by and from the members of the Panel Committee, two members appointed by the Insurance Committee, one of whom should be a member of the Committee representative of insured persons, and one a registered pharmacist, and, in addition, a practitioner to be appointed by the sub-committee who should act as their chairman. In this way it is pointed out that a medical majority is assured with a medical chairman.

"I think," says Mr. Lee Gordon, "that all who have had the opportunity of hearing doctors discussing formulæ and dispensing questions will realise that they positively welcome the competence and experience in such matters which a qualified chemist, if present, will import into the discussion. As to the private member, it is reasonable to assume that his knowledge of the general principles involved, his administrative experience and his fund of worldly lay common-sense, will prove a wholesome ingredient in this small mixed body."

**Testing of Medicines.**.—Mr. W. Shaw, the clerk to the St. Helens Insurance Committee, read a paper on this subject which was particularly valuable, because it described the procedure actually in operation in his area in testing medicines to see if they are accurately compounded in accordance with the prescriptions. Distinction was drawn in this paper between a scheme of testing whereby the Medical Officer of Health took samples on prescriptions written by insurance practitioners acting in co-operation with the Insurance Committee, and then took proceedings under the Food and Drugs Act, and a purely Insurance Committee scheme. Under such a scheme the Clerk arranged for taking samples, their analysis, and the subsequent consideration of the analyst's reports by a specially constituted sub-committee, who would either caution the chemist, where any irregularity appeared to be slight, or refer the matter to the sub-committee responsible under the regulations for carrying out formal procedure with a view to appropriate penalties.

There is no doubt that the Insurance Committee can quite well keep the machinery for these medicinal tests in their own hands—subject, perhaps, to the co-operation of the local authority in regard to the taking of samples by their officers, acting as agents of the Insurance Committee—and that their judicial procedure is better suited for dealing with cases of irregularity than is the Police Court procedure under the Food and Drugs Acts. The range of penalty which the Insurance Committee have it in their power to recommend, from the withholding of small or large sums of Exchequer grant from the chemist, to removal from the panel, is much wider; on the other hand, there may be isolated cases of error, where the publicity of police court proceedings would unfairly penalise the chemists. What is perhaps more important is the presence of chemists themselves on the Insurance Committee tribunals; and we have no hesitation in saying that the chemists as a body are the first to welcome a wise and reasonable use by the Insurance Committees of the method which is at their disposal for testing supplies. A sum of £1,500,000 is being spent on medicines and appliances for the insured in England and Wales; it rests with the Insurance Committees to see that the goods supplied are those which are ordered.

**Nursing the Insured.**—The letter from the Cheshire Insurance Committee which appears in this issue seeks to revive a question which agitated Insurance Committees a year ago, and is at the moment more or less quiescent; as to its importance there is no doubt, and we hope to refer more fully to the subject in our next issue.

